# Prediction of the efficacy after the first transarterial chemoembolization in hepatocellular carcinoma using CT radiomics combined with inflammatory composite indicators

**DOI:** 10.3389/fmed.2026.1733481

**Published:** 2026-05-21

**Authors:** Jia hao Sun, Yi xiong Zhang, Bo sheng Luo, Jia ping Wang

**Affiliations:** 1 Department of Radiology, The Second Affiliated Hospital of Kunming Medical University, Kunming, China; 2 Radiology Department of the Second Affiliated Hospital of Kunming Medical University, Kunming, China

**Keywords:** hepatocellular carcinoma, inflammatory composite indicators, radiomics, therapeutic efficacy, transarterial chemoembolization

## Abstract

**Background:**

Hepatocellular carcinoma (HCC) ranks among the most prevalent tumors globally. Transcatheter arterial chemoembolization (TACE) serves as the standard treatment for intermediate and advanced stages of HCC. However, patient responses to TACE vary significantly. This study aims to assess the predictive value of combining CT radiomics with inflammatory composite indicators for evaluating the efficacy of initial TACE in HCC patients.

**Methods:**

We included 175 patients with pathologically confirmed HCC, categorizing them into a good efficacy group (95 cases) and a poor efficacy group (80 cases). We compared radiomics features and inflammatory composite indicators between these groups. To identify independent risk factors for predicting TACE efficacy, we performed multivariate Logistic regression analysis. We developed a radiomics prediction model and a clinical prediction model based on inflammatory composite indicators. A combined prediction model was created using selected inflammatory composite indicators and radiomics features, and visualized with a nomogram. We assessed the model's predictive performance using the receiver operating characteristic (ROC) curve, and its stability and authenticity through 1,000 bootstrap resampling. The clinical benefit was evaluated using a decision curve analysis (DCA) curve.

**Results:**

The multivariate logistic regression analysis revealed that platelet-to-lymphocyte ratio (PLR), maximum tumor diameter, and Radiomics score (Radscore) were independent risk factors for predicting the efficacy of the first TACE in HCC. The clinical model, based on the inflammatory composite index, achieved an AUC of 66.6 for efficacy prediction. The radiomics model, developed from radiomics features, demonstrated an AUC of 76.1. Notably, the combined prediction model, integrating both radiomics features and the inflammatory composite index, achieved an AUC of 80.4.

**Conclusion:**

CT radiomics, when combined with composite inflammatory indicators, demonstrated high predictive efficacy for the first TACE treatment outcomes in HCC patients. The developed visual nomogram aids clinicians in creating personalized pre-operative treatment plans for these patients.

## 1 Introduction

Hepatocellular Carcinoma (HCC) ranks as the sixth most common cancer and the third leading cause of cancer-related deaths worldwide, highlighting its significant incidence and mortality rates (1, 2). Transcatheter Arterial Chemoembolization (TACE) is the standard treatment for intermediate and advanced stages of HCC. However, its efficacy varies significantly among individuals (3). Studies indicate that the objective response rate to TACE differs among patients, with conventional TACE achieving an approximate 50% response rate (4). Additionally, some patients may develop resistance to TACE (5). Therefore, there is an urgent need for precise predictive tools to guide personalized treatment strategies.

Radiomics involves extracting and analyzing quantitative data from medical images. This evolving field transforms standard medical images into high-dimensional, mineable data using automated or semi-automated quantitative analysis methods. These methods uncover deep subvisual information related to pathology, immunophenotype, and the tumor microenvironment, the goal is to develop novel biomarkers (6), it has currently demonstrated potential in predicting the efficacy of TACE for HCC (7). However, a single imaging feature cannot fully capture the heterogeneity of HCC, including the biological complexity of the tumor microenvironment. Studies indicate that radiomic signals may correlate with specific immune microenvironments (8). Additionally, inflammation-related cells and factors in the tumor microenvironment can inhibit immune responses (9), with the inflammatory process playing a crucial role in liver cancer progression and treatment resistance.

Inflammatory composite indicators serve as crucial tools that reflect the dynamic balance between chronic inflammation and immune regulation within the tumor microenvironment and possess significant clinical value (10). The occurrence and progression of liver cancer closely correlate with chronic inflammation. The inflammatory microenvironment can promote tumor progression while also potentially activating anti-tumor immune responses (11). The PLR serves as a composite inflammatory marker that evaluates the body's systemic inflammatory status by integrating platelet and lymphocyte counts. As a comprehensive indicator of inflammation and immune response, it demonstrates significant value in the prognostic assessment of various cancers. This association likely arises from the central role of chronic inflammation in the occurrence and development of HCC. The inflammatory microenvironment drives liver cancer progression by promoting immune escape and tumor cell proliferation (12, 13). Importantly, as a non-invasive biomarker, changes in PLR levels can also indicate the potential responsiveness of HCC patients to immunotherapy (14). Collectively, this evidence suggests that PLR may serve as an auxiliary indicator for prognostic stratification and individualized treatment of HCC patients (15).

The field of liver cancer research is currently focused on exploring the application of combined models to address the complex pathological mechanisms and treatment challenges of liver cancer, particularly HCC. Evidence suggests that incorporating clinical characteristics or biological markers can improve prediction model performance (16). However, there is a need for more integrated approaches. Few studies have investigated the efficacy prediction of the first TACE in HCC patients by combining CT radiomics, clinical characteristics, and inflammatory composite indicators. This study aimed to assess the predictive value of preoperative radiomic features, clinical data, and inflammatory composite indicators for the efficacy of the first TACE in HCC patients. We also sought to develop a clinical prediction model using a nomogram. This model would convert radiomic features, inflammatory indicators, and clinical data into a visual tool, enhancing patient stratification and personalized management.

## 2 Material and methods

### 2.1 Study population

Our study received ethical approval from the institutional ethics committee. We conducted a retrospective analysis of CT imaging and clinical data from 175 patients diagnosed with HCC at our hospital between January 2016 and August 2024, all of whom received TACE as their initial treatment. Inclusion criteria were as follows: ① Diagnosis of HCC confirmed by liver biopsy; ② Underwent a three-phase contrast-enhanced CT scan within 2 weeks prior to treatment; ③ No evidence of extra-hepatic metastasis; ④ TACE used as the first-line treatment. Exclusion criteria included: ① Presence of other liver diseases (except hepatitis B) or heart, brain, and kidney diseases; ② Drug addiction and/or HIV infection; ③ Other organ malignancies; ④ Lesions with enhanced parts smaller than 5 mm; ⑤ Lesions comprising more than 75% of total liver volume; ⑥ Prior systemic or local anti-tumor treatments (e.g., chemotherapy, surgical resection, immunotherapy, and ablation) before TACE; Poor quality contrast-enhanced CT images, significant respiratory motion artifacts, or inability to delineate the region of interest in different phases.

### 2.2 Inspection methods and surgical procedures

All preoperative CT scans were conducted using the Brilliance iCT scanner by Philips, Netherlands. Images chosen for analysis were from the late arterial phase, characterized by clear enhancement of the hepatic artery and initial enhancement of the portal vein, occurring approximately 24–32 s after contrast agent injection. The late arterial phase is the peak enhancement phase for liver cancer lesions. During this phase, tumor tissue demonstrates pronounced enhancement because of adequate hepatic arterial blood supply. Radiomic features from this phase directly reflect tumor blood supply, proliferative activity, and microvessel density—key predictors of TACE efficacy.

All TACE procedures were conducted using the Syngo Axiom-Artis DSA machine from Siemens AG, Germany. Each operating physician had over 8 years of experience in abdominal interventional procedures. The Seldinger technique was employed for femoral artery puncture. For angiography of the celiac trunk and superior mesenteric arteries, either a 5F-RH or 5F-YASHIRO catheter was utilized to determine the tumors' location, size, number, and feeding vessels. A microcatheter facilitated superselective catheterization into the tumor's feeding artery. Oxaliplatin (50–100 mg) and fluorouracil (500–1,000 mg) were administered slowly through the microcatheter for transcatheter arterial infusion chemotherapy, with an infusion duration of at least 20 min. Following the infusion, transcatheter arterial embolization of the tumor's feeding artery was performed. Common embolic materials included lipiodol with pirarubicin suspension, polyethylene embolic microspheres, and gelatin sponge particles. The embolization endpoint was achieved with the occlusion of the tumor's feeding artery and the disappearance of tumor staining. Post-procedure, patients routinely received liver protection, anti-vomiting, and analgesic treatments.

### 2.3 Postoperative Efficacy Assessment

One month after TACE, the patient underwent a three-phase enhanced CT reexamination. Postoperative efficacy was assessed using the mRECIST (modified response evaluation criteria in solid tumors) standard. The mRECIST criteria are specifically developed for the efficacy evaluation of local liver treatments and serve as the core basis for efficacy determination in this field.A complete response (CR) indicated the disappearance of arterial enhancement in all target tumor lesions, no new lesions, and normal tumor markers. A partial response (PR) was defined as a reduction of at least 30% in the sum of the diameters of the target lesions compared to the baseline. Stable disease (SD) was assigned to cases that did not qualify as remission or progressive disease. Progressive disease (PD) involved an increase of at least 20% in the sum of the maximum diameters of the target lesions compared to the study period's minimum sum, or the appearance of new lesions. CR and PR were categorized as the good efficacy group, while SD and PD were classified as the poor efficacy group.

### 2.4 Tumor segmentation, feature extraction and selection

All preoperative images of patients were stored in DICOM format and anonymized. Using 3Dslicer software (Version 5.9.0, www.slicer.org), experienced radiologists delineated the contours of the target region of interest (ROI) layer by layer on the late arterial phase CT images. Open-source PyRadiomics 3.10 software then extracted imaging features from these ROIs, including shape, first-order, texture, Gaussian filtering, and wavelet features. One month later, another pair of radiologists re-delineated the ROIs for 30 randomly selected patients to assess intra-observer consistency. We evaluated this using the inter-observer correlation coefficient (ICC), retaining radiomics features with ICC ≥ 0.8 for further analysis. Preliminary screening employed the variance method and *t*-test, followed by Z-score normalization of the data. The least absolute shrinkage and selection operator (LASSO) method was then used to select the optimal features.

### 2.5 Model construction and evaluation

Clinical indicators and inflammatory composite indicators with significant statistical differences were selected for logistic regression analysis to identify independent risk factors and develop a clinical combined model. Optimal radiomics features, selected using LASSO, were used to calculate the Radscore and construct a radiomics model. These clinical features, inflammatory indicators, and radiomics features were integrated to establish a combined model. Each model's performance was assessed using the receiver operating characteristic (ROC) curve, and the area under the curve (AUC) was calculated. To test the model's stability and authenticity, a 1,000-bootstrap resampling method was employed. Decision curve analysis (DCA) evaluated the model's clinical utility.

### 2.6 Statistical analysis

In this study, we conducted statistical analyses using SPSS 27.0 software. The Shapiro-Wilk test determined if continuous variables followed a normal distribution. When data were normally distributed with homogeneous variances, we applied the independent samples *T*-test. For normally distributed data with heterogeneous variances, Welch's *T*-test was employed. If data did not follow a normal distribution, the rank-sum test was utilized. Categorical variables were analyzed using the chi-square test. We used Python software (Version 4.2.2) to generate calibration curves, DCA curves, and nomograms. *P*-value of less than 0.05 indicated statistical significance.

## 3 Results

### 3.1 Characteristics of the study population

All the data were obtained from the Second Affiliated Hospital of Kunming Medical University, and the data were complete without any missing values. This study included 175 patients with HCC, categorized based on the mRECIST criteria into a good-efficacy group (CR + PR, *n* = 95) and a poor-efficacy group (SD + PD, *n* = 80). Statistical analysis indicated significant differences between the groups concerning SIRI, SII, NLR, PLR, and maximum tumor diameter (*P* < 0.05; Table 1). These statistically significant variables were subjected to univariate logistic regression analysis. The results identified SII, PLR, and maximum tumor diameter as significant factors influencing the efficacy of the first TACE in HCC patients (*P* < 0.05; Table 2).

**Table 1:** Comparison of inflammatory composite indicators and clinical characteristics between the group with good curative effect and the group with poor curative effect.

Variables	Total (*n* = 175)	good-efficacy (*n* = 95)	Poor-efficacy (*n* = 80)	*P*-value
Sex				0.5540
Male	149	79	70	
Female	26	16	10	
Age				0.5360
≤ 60	105	55	50	
>60	70	40	30	
AFP	83.110 (8.090, 530.265)	63.114 (8.690, 304.990)	139.090 (7.690, 1,000.000)	0.4280
TBIL	17.900 (13.000, 26.600)	18.400 (13.900, 28.300)	17.450 (12.200, 22.850)	0.0980
PT	13.100 (12.100, 14.450)	13.200 (12.000, 14.450)	13.100 (12.175, 14.425)	0.9280
SIRI	0.971 (0.555, 1.720)	0.861 (0.536, 1.404)	1.116 (0.670, 2.112)	0.04148
SII	371.874 (200.417, 713.301)	288.600 (176.208, 520.770)	499.979 (242.166, 892.186)	0.00048
NLR	2.597 (1.738, 4.005)	2.240 (1.552, 3.433)	2.937 (1.934, 4.127)	0.02186
PLR	117.606 (88.522, 170.081)	107.576 (83.233, 141.542)	135.314 (101.182, 193.302)	0.00085
MLR	0.328 (0.222, 0.463)	0.323 (0.220, 0.438)	0.345 (0.225, 0.508)	0.62116
Maximum.tumor.diameter	8.000 (5.000, 11.100)	6.600 (4.650, 9.650)	9.600 (6.000, 12.537)	0.00072
Child classification				0.6690
A	112	63	49	
B	60	30	30	
C	3	2	1	

AFP, alpha fetoprotein; TBIL, total bilirubin; PT, Prothrombin Time; SIRI, Systemic Inflammation Response Index; SII, Systemic Immune-Inflammation Index; NLR, neutrophil-to-lymphocyte ratio; PLR, platelet-to-lymphocyte ratio; MLR, monocyte-to-lymphocyte ratio.

**Table 2:** Univariate logistic regression analysis of inflammatory composite indicators and radiomics features.

Variables	β	S.E	*Z*	*P*	OR (95%CI)
SIRI	0.09	0.09	1.09	0.276	1.10 (0.93 ~ 1.30)
SII	0.01	0.00	2.62	0.009	1.01 (1.01 ~ 1.01)
NLR	0.03	0.05	0.52	0.606	1.03 (0.92 ~ 1.15)
PLR	0.01	0.00	2.82	0.005	1.01 (1.01 ~ 1.01)
MLR	0.08	0.62	0.13	0.897	1.08 (0.32 ~ 3.67)
Maximum.tumor.diameter	0.13	0.04	3.26	0.001	1.14 (1.05 ~ 1.24)
Radscore	2.66	0.50	5.29	< 0.001	14.32 (5.34 ~ 38.41)

S.E, standard error; 95%CI, 95% confidence interval.

### 3.2 Radiomics features

**Figure 1:**
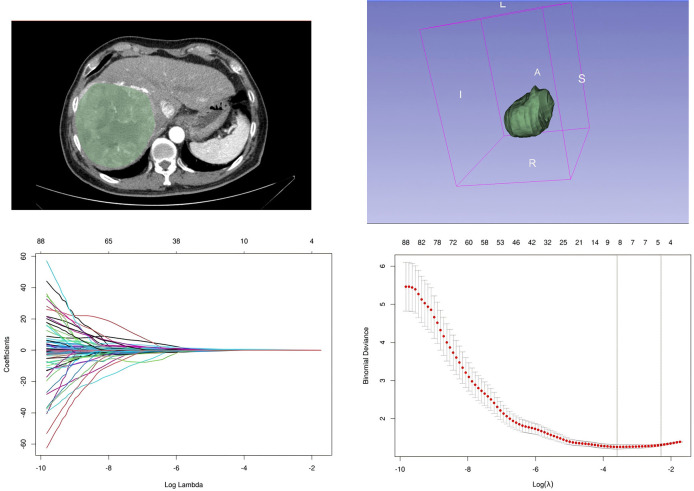
ROI delineation and feature selection by LASSO regression.

A total of 1,621 features were initially extracted from the CT images during the late arterial phase. Following preliminary screening with the variance method and rank-sum test, 122 features were retained. These selected features were then subjected to LASSO regression analysis (Figure 1). Ultimately, 8 radiomics features were identified, and their corresponding weights and Radscore were calculated (Table 3). When Radscore was incorporated into the univariate logistic regression analysis, the results indicated that Radscore significantly influenced the efficacy of the first TACE in HCC patients (*P* < 0.05; Table 2).

**Table 3:** The finally selected radiomics features and their coefficient values.

Filter	Feature class	Feature name	Coefficient
wavelet-LHH	gldm	Gray level non uniformity	0.177596496
wavelet-HHH	glszm	Large area high gray level emphasis	0.352274537
wavelet-LLH	glcm	MCC	0.519636015
log-sigma-2.0mm-3D	glszm	Small area high gray level emphasis	0.005297992
original	shape	Major axis length	0.128363742
original	firstorde	Range	0.056562754
original	ngtdm	Complexity	0.068246762
original	diagnostics	Maximum	0.085245603

### 3.3 Efficiency analysis of different models

Model 1, a clinical model, was developed by integrating three indicators: SII, PLR, and maximum tumor diameter. These were selected based on univariate logistic regression analysis results. Model 2 is a radiomics prediction model, created by incorporating Radscore into logistic regression (Table 3).

**Figure 2:**
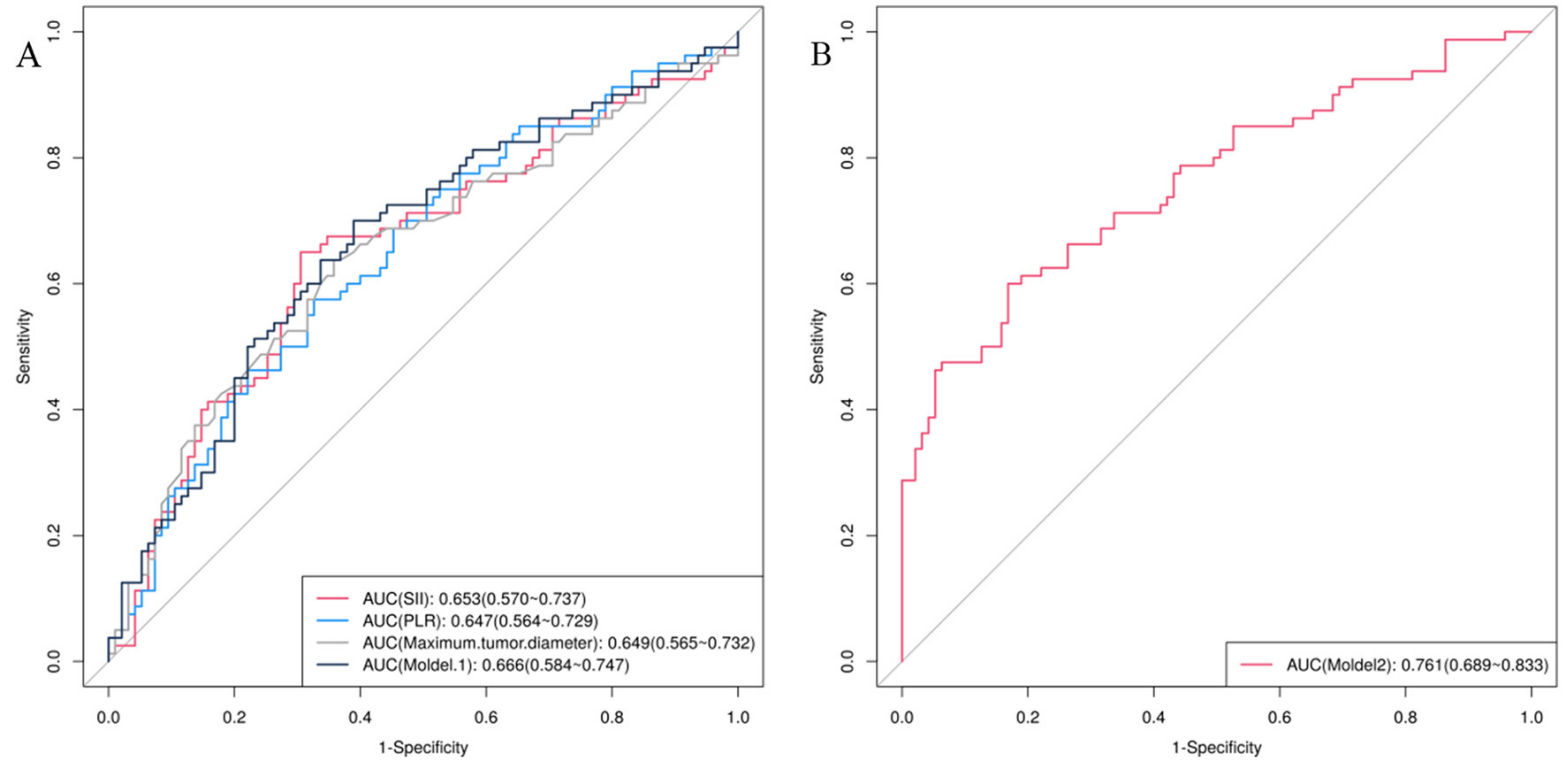
Results of ROC curve analysis for model 1**(A)** and model 2**(B)**.The predictive efficacy of model 1 is higher than that of each independent predictor.

Model 1 integrated the inflammatory composite index with clinical features, resulting in an AUC value of 66.6 (Figure 2A). In contrast, Model 2 incorporated radiomics features, yielding an AUC value of 76.1 (Figure 2B).

We included SII, PLR, Radscore, and maximum tumor diameter in the multivariate logistic regression analysis. Recognizing the theoretical high correlation between SII and PLR, we assessed their collinearity. The variance inflation factor was 3.26, indicating moderate collinearity. To mitigate potential impacts on model stability and enhance interpretability, PLR and SII are incorporated separately into the multivariate logistic regression. The results indicate that the *p*-value for PLR is < 0.05, while the *p*-value for SII is > 0.05 (Table 4). Ultimately, the study concludes that PLR, Radscore, and maximum tumor diameter serve as independent risk factors for predicting the efficacy of the first TACE in HCC patients (Table 5).

**Table 4:** Multivariate logistic regression analysis of inflammatory composite indicators and radiomics features.

Variables	β	S.E	*Z*	*P*	OR (95%CI)
SII	0.001	0.00	2.91	0.088	1.001 (1.000 ~ 1.001)
Maximum.tumor.diameter	0.14	0.07	4.72	0.030	0.866 (0.761 ~ 0.986)
Radscore	3.51	0.73	23.19	< 0.001	33.39 (8.01 ~ 139.24)

S.E, standard error; 95%CI, 95% confidence interval.

**Table 5:** Multivariate logistic regression analysis of inflammatory composite indicators and radiomics features.

Variables	β	S.E	*Z*	*P*	OR (95%CI)
PLR	0.01	0.00	2.37	0.018	1.01 (1.01 ~ 1.01)
Maximum.tumor.diameter	0.17	0.07	2.47	0.0013	0.84 (0.74 ~ 0.97)
Radscore	3.71	0.76	23.99	< 0.001	40.86 (9.26 ~ 180.29)

S.E, standard error; 95%CI, 95% confidence interval.

**Figure 3:**
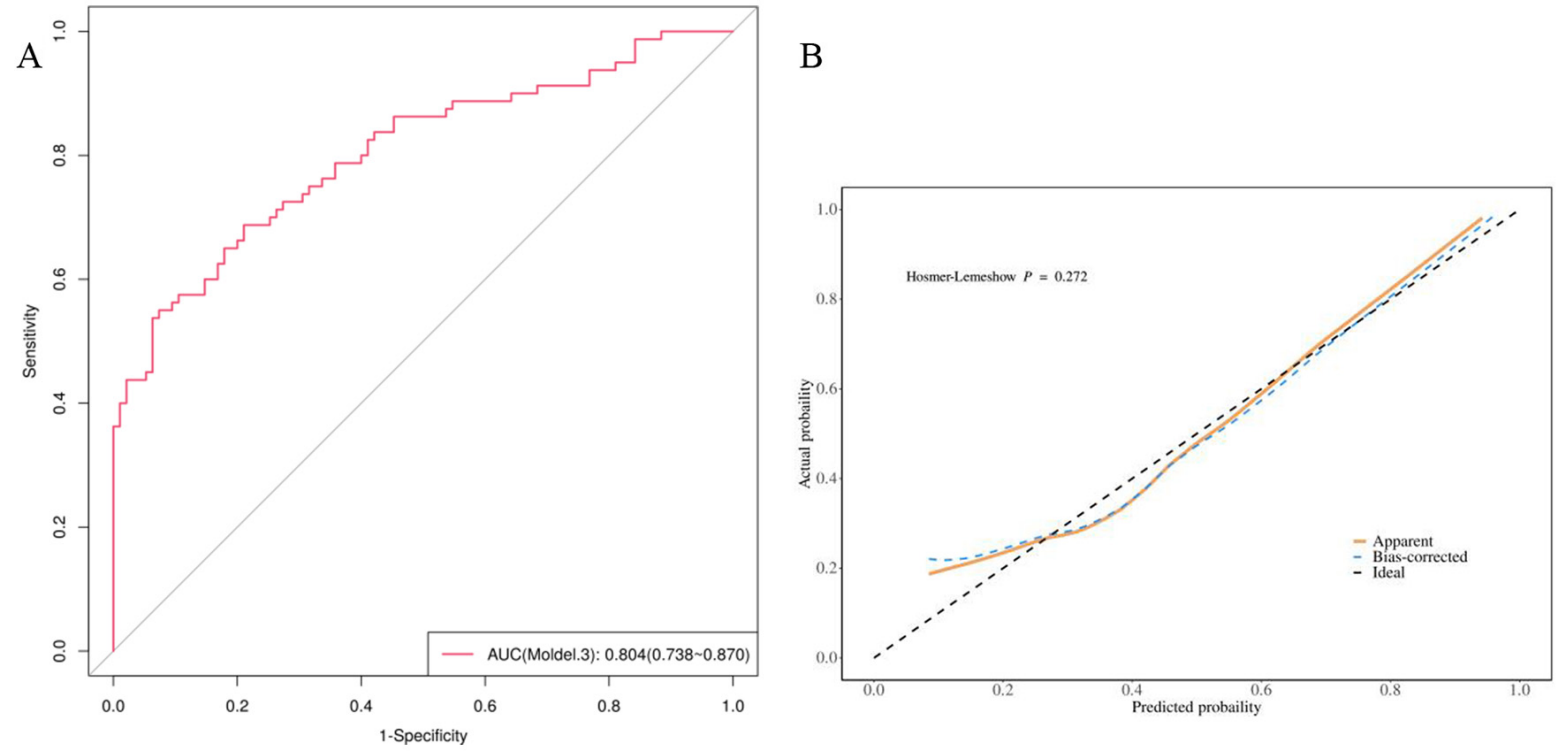
**(A)**The ROC curve of model 3; **(B)**The calibration curve of Model 3 shows that there is a good consistency between the predicted TACE efficacy and the actual TACE efficacywith dotted line (actual calibration) closed to dashedline (perfect calibration).

Three indicators—PLR, Radscore, and maximum tumor diameter—were used to develop a combined model, referred to as Model 3. This model integrated inflammatory composite indicators, clinical characteristics, and radiomics features. Model 3 achieved an AUC value of 80.4, surpassing those of Model 1 and Model 2, demonstrating its strong predictive capability. To validate the model, a calibration curve was generated using the 1,000 bootstrap resampling method. The *P*-value from the Hosmer-Lemeshow test was 0.272 (*P* > 0.05), indicating no statistically significant difference and suggesting a good model fit (Figure 3).

**Figure 4:**
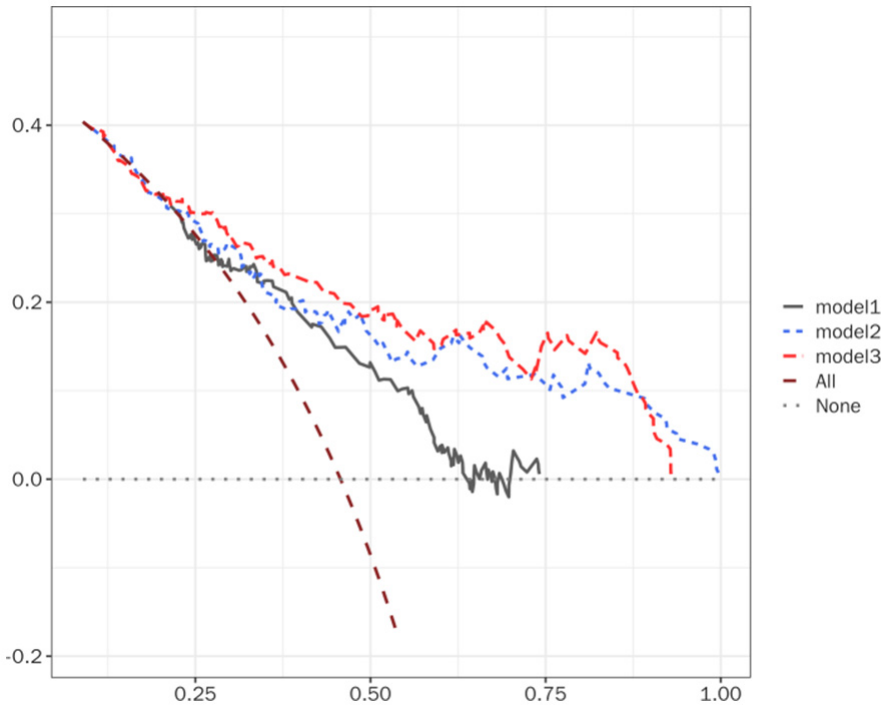
Decision curves for model 1, model 2, and model 3.

We evaluated the performance of all models using the DCA curve. The results indicated that Model 3 had the largest area under the decision curve, demonstrating the greatest benefit across a broad range of threshold probabilities (Figure 4).

### 3.4 Nomogram construction

**Figure 5:**
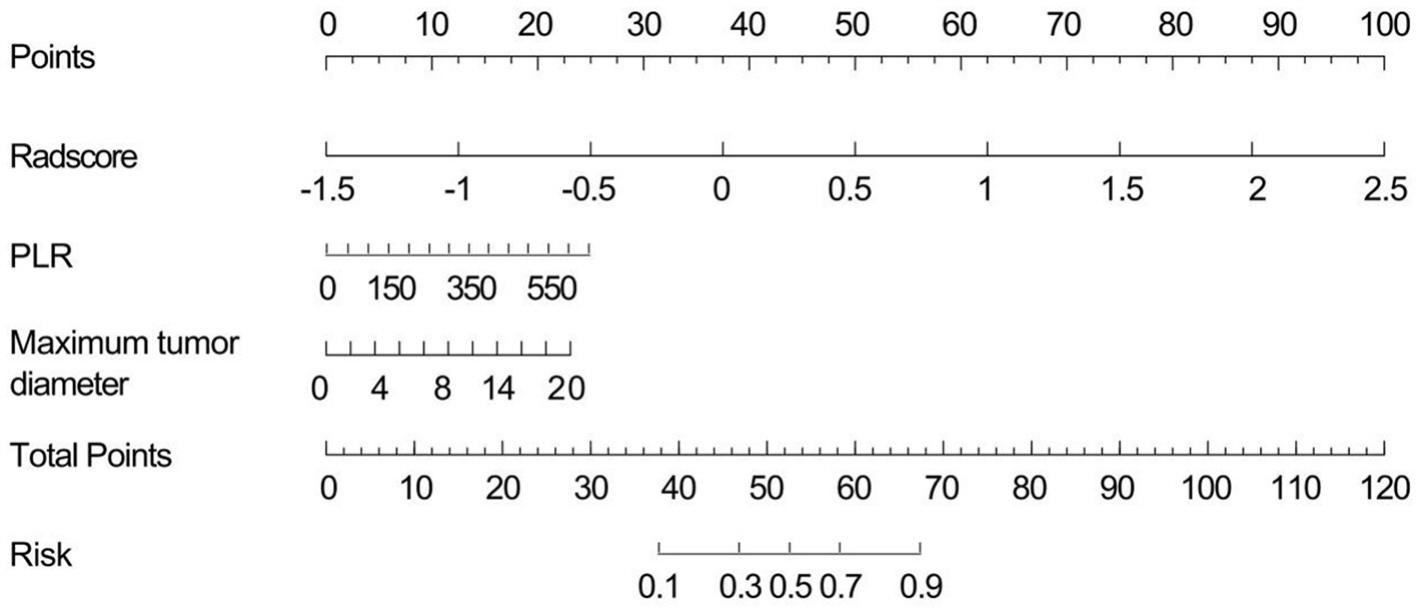
Visualized nomogram model.

We developed a nomogram prediction model to assess the efficacy of the first TACE in patients with HCC (Figure 5). This model incorporates three key indicators: the PLR, Radscore, and the maximum tumor diameter. By analyzing the visual output of the model, we evaluated the relationship between the total score of these predictive variables and the effectiveness of TACE.

## 4 Discussion

This study evaluates the effectiveness of combining CT-based radiomics features with inflammatory composite indicators to predict the success of the initial TACE treatment in patients with HCC. The findings reveal that both the radiomics and clinical models individually offer some predictive capability for TACE outcomes in HCC patients. However, the integrated prediction model, which combines these multi-dimensional indicators, demonstrates superior predictive performance. Its AUC value is significantly higher than that of any single model, indicating substantial added value. Additionally, the DCA shows favorable clinical benefits, underscoring its practical applicability.

In liver cancer research, radiomics has become a promising non-invasive tool, providing objective evidence for clinical decision-making (17). It transforms imaging data into a high-dimensional feature space to reveal biological insights like tumor heterogeneity and microenvironment, which are not visible to the naked eye (7, 18). In this study, four of the radiomics features are texture features, while the remaining four are first-order and shape features. Texture features utilize mathematical matrices to describe the spatial dependence and heterogeneity of gray values within a local image region, highlighting the internal microstructural differences of lesions. These features address the limitations of first-order statistical and shape features, offering a more comprehensive perspective for image analysis (19).

Recent studies indicate that the effectiveness of TACE for HCC is linked to the host's inflammatory response. Local inflammation within the tumor microenvironment plays a crucial role in tumor development (20). Research by Tan J et al. revealed that post-TACE, the number of CD8+ T cells in the microenvironment decreased, while tumor-associated macrophages significantly increased, particularly the subset with high TREM2 expression. This immunosuppressive environment correlates with tumor recurrence and reduced TACE efficacy (21). Consequently, inflammation has become a focal point in recent research on TACE's impact.

Our research demonstrated that PLR serve as independent risk factors for predicting the efficacy of the first TACE in HCC. The systemic inflammatory response can impact tumor progression by inhibiting apoptosis, promoting angiogenesis, and causing DNA damage. As markers of systemic inflammation, elevated PLR may suggest a pro-tumor inflammatory state, potentially linked to an increased risk of residual lesions or early recurrence post-TACE. Rocco A et al. investigated the predictive capability of combining SII, PLR, and other inflammatory indicators, such as NLR, for HCC prognosis. They found that SII and PLR retained independent significance in multivariate analysis, particularly in recurrence risk stratification (22). Öcal et al. confirmed through a multicenter randomized trial that PLR possesses predictive value for the combined treatment of advanced HCC using selective internal radiation therapy (SIRT) and sorafenib (23). These findings imply that PLR are reliable indicators of tumor biological behavior, and their predictive value may extend to evaluating TACE treatment efficacy, aligning with our multivariate logistic regression results.

In this study, the radiomics feature model demonstrated superior predictive efficacy (AUC value: 76.1) compared to the clinical model (AUC value: 66.6). This enhanced performance of the radiomics model may result from its capacity to capture microscopic features like tumor heterogeneity, which are challenging to obtain through conventional clinical parameters (24). Radiomics quantitatively analyzes texture and shape features in CT/MRI images, reflecting variations in the tumor microenvironment (25). A study by Hapaer G et al. successfully predicted the efficacy of PD-1 inhibitors using radiomics features, highlighting its ability to supplement biological information not accessible through clinical indicators (26). The clinical model's relatively lower performance may stem from its reliance on macroscopic measurements, which lack sensitivity to microvascular changes following TACE treatment (27). Nonetheless, the clinical model remains valuable due to its strong interpretability and ease of access, making it an essential component of model construction (28).

In our study, we integrated radiomics features, inflammatory composite indicators, and clinical features to predict the efficacy of the first TACE in patients with HCC. This integrated model significantly outperformed both the individual radiomics feature model and the clinical combination model. The improvement likely results from the complementary nature of clinical variables and radiomics features, which together capture the complexity of tumor biological behavior (29, 30). Additionally, we constructed a nomogram to visualize the combined model, clearly illustrating the various risk factors affecting TACE efficacy. Based on individualized scoring of different patient conditions, we conducted risk stratification and prognosis grading.The study by Qiao W et al. developed a nomogram for patients with BCLC stage A/B HCC who underwent TACE combined with ablation therapy. This tool accurately predicted 1-year, 3-year, and 5-year recurrence-free survival and stratified patients into different recurrence risk groups, allowing for timely intervention for high-risk individuals (31). Liang et al.'s study involved HCC patients who had radical resection, using a multi-center database to construct two nomogram models for those who received or did not receive adjuvant TACE. These models incorporated eight independent predictors, including portal hypertension, Child-Pugh score, and AFP level. They were validated using the C-index and calibration curves, effectively quantifying the expected survival benefit of adjuvant TACE (32). Collectively, these studies have demonstrated that the nomogram serves as an effective visual clinical tool. It employs regression analysis to identify key risk factors and provides individualized predictions through a graphical interface. Currently, nomograms are applied in predicting various diseases (33, 34).

Our study faces several limitations: ① As a single-center retrospective study, our findings require validation through multi-center and prospective research. ② We focused solely on late arterial phase images for radiomics analysis. ③ The study's relatively small sample size may restrict our ability to control for potential confounding factors, potentially impacting the results.

In conclusion, the combined model based on CT radiomics and inflammatory composite indicators demonstrated high predictive efficacy for assessing the first TACE treatment's effectiveness in HCC patients. The developed visual nomogram aids clinicians in crafting personalized pre-operative treatment plans for these patients.

## Data Availability

The original contributions presented in the study are included in the article/supplementary material, further inquiries can be directed to the corresponding author.
